# Knowledge and factors associated with pain management for hospitalized children among nurses working in public hospitals in Mekelle City, North Ethiopia: cross sectional study

**DOI:** 10.1186/s13104-017-2446-7

**Published:** 2017-03-09

**Authors:** Remla Miftah, Weyzer Tilahun, Atsde Fantahun, Seid Adulkadir, Kahsu Gebrekirstos

**Affiliations:** 1 Dr. Tewelde Health Science College, Mekelle, Tigray Ethiopia; 2 Department of Nursing, College of Health Science, Mekelle University, Mekelle, Tigray Ethiopia; 3 Department of Physiotherpy, Ayder Referral Hospital, Mekelle University, Mekelle, Tigray Ethiopia

**Keywords:** Knowledge, Pain management, Hospitalized children, Nurses, Associated factors

## Abstract

**Background:**

American Nurses Association reflects, the role of the nurse in pain management encompasses the entire nursing process, assessment of pain, plans pharmacological and non-pharmacological pain management strategies, implements the plan, and evaluates the response of the patient to the interventions. Pediatric pain management has been left largely unaddressed due to factors like limited resources, inadequate training, as well as cultural diversity and language barriers which made sick and injured children not to receive basic pain care. The objective of this study was to assess the knowledge and factors associated with pain management for hospitalized children among nurses.

**Design:**

Institution based cross-sectional study was employed from a total of 261 nurses in Public Hospitals of Mekelle City from March 15 to April 15, 2015. Systematic random sampling method was used to get the study subjects. Self-administered questionnaire was used to collect data. The collected data was cleared, categorized, and coded. The cleaned data was analyzed using Statistical Package for the Social Sciences, version 20 software with statistical significance p < .05 at 95% CI. Descriptive statistics was employed. Binary logistic regressions were used to see relationship between dependent and independent variables.

**Results:**

Out of 251 participants more than half (58.6%) of nurses had adequate knowledge and had good practice 140 (55.8%). Those respondents who said yes sedation interfering with pain assessment were 2.7 more likely knowledgeable on pain management for hospitalized children than others. In addition to this those nurses who said they had a specific pain management protocol in their institution were 2.159 more likely knowledgeable than others.

**Conclusions:**

Majority of nurses were knowledgeable on some of pharmacological and non-pharmacological pain managements. Most of the nurses had a good practice on children pain managements. Reading guide lines, specific protocols, knowledge, charting area for pain, sedation interfering with pain assessment and working in pediatric ward were some of the factors that were significantly associated with children’s pain management.

## Background

Pain is unpleasant emotional and sensory complain associated with actual or potential tissue damage as defined by International Association for the Study of pain [1]. Since the late 1980s, children’s pain has become recognized as an important clinical concern and main area of focus for different researchers [1]. In the contrary, pain management services are not easily available to children in the developing world, where there is difficulty to give standard care [2]. In 2009, it was estimated that more than 33 million Americans were living with serious pain that lasted 1 year or more [3]. Different hospitals have taken numerous steps to improve their pain management strategies and Joint Commission standards indicate in 2003 patients have a right to effective pain management and routine pain assessment for all patients [3].

According to the American Nurses Association (ANA), the role of nurses in pain management includes the entire nursing process, assessment of pain, plan of pharmacologic and non-pharmacologic pain management strategies, implementation and evaluation of the response of the patient to the interventions [1]. Non-pharmacologic approaches to treat pain in children include psychological, educational and parental support [4]. For children undergoing frequent painful procedures, cognitive-behavioral interventions that minimize anxiety and distress, can be quite effective [4]. Non steroidal anti-inflammatory drugs (NSAIDS) like aspirin are very effective for the management of mild-to moderate pain or in combination with opioids (e.g. codeine, pethidine) which are commonly given in health institutions in the study area)for more severe pain [4]. The advantages of good analgesia are particularly important for ill patients because of its physiologic and psychologic benefits [5]. Adequate pain management reduces anxiety of children and parents and increases compliance and collaboration, which can reduce some of the burden on medical staff and resources [6]. In light to this evidence, proficient pain management for ill patients is a significant factor in meeting their needs and maximizing the chance of recovery [7]. Inadequately treated acute pain has a negative impact on many systems of the body, harmful physical and psychologic consequences in all age group clients [5].

Eventhough increased effort has been used to improve pain management over the last decade, up to 81% admitted children complain moderate to severe levels of pain [8]. However, nurses administer only 23–43% of analgesics ordered [9]. Pediatric pain management has been left largely unaddressed due to factors like limited resources, inadequate training, as well as cultural diversity and language barriers which made sick and injured children not to receive basic pain care [10]. Nurses’ knowledge and attitudes can affect their ability to give effective pediatric pain management [11]. In addition to that personal values and beliefs of the health care providers about pain greatly affect the treatment of pain [11]. For instance, 55–90% nurses believe that children exaggerate their pain report [11]. Lack of knowledge about Opioid, negative attitudes toward prescribing Opioid, and inadequate pain-assessment skills, lack of communication skills combine to create major barriers to pain relief [12]. Therefore, the main objective of this study was to assess the knowledge and factors associated with pain management for hospitalized children among nurses working in public Hospitals in Mekelle City, North Ethiopia.

## Methods

This institution based cross sectional study was conducted in Mekelle City; public hospitals from March 15 to April 15, 2015. All Nurses who had been working in all Public Hospitals of Mekelle City were the source population. The total sample size was 261 nurses which was calculated using single population proportion formula with the assumption of proportion of nurses who had sufficient knowledge on pain management (41%), with 5% margin of error and 95% confidence level of certainty. There are four public hospitals in Mekelle City, lottery method was used to select three public hospitals in the city. A list of all the four hospitals was prepared and it was drawn blindly to select the three hospitals. After proportional allocation to sample size to each hospital systematic random sampling method was used to select study participants with K value of 2 (meaning, every other nurse is selected). Simple random sampling had been employed to select the first study subjects. The data collection tool was English version structured questionnaire developed from different literatures because the authors cannot find standardized questionnaire specifically in children. The questionnaire had 3 parts: part one sociodemographic of respondents; part two Knowledge related questions and part 3 factors associated with pain management related questions. The questions were reviewed by experts and seniors. Three trained diploma nursing students and one degree nurse supervisor was recruited and participated throughout the data collection procedures. Before the actual data collection period, to ensure the validity and reliability of the questionnaire, it had been pre-tested on 5% of nurses working in 1 non-selected public hospital of the city.

The filled questionnaires were presented to the principal investigator every day and had been checked for its completeness. Responses in each question had been coded to make the data entry simpler. SPSS version 20 software was used for data analysis. Descriptive analysis, such as, frequency and measures of central tendency was computed as well as bivariate analysis had been used to assess the association between the dependent and independent variables. Then the assumptions of, interaction, multi co-linearity, and outliers where checked and considering the p < .05 cut off point as significant for all the independent variables, the model logistic regression was computed. Then the result had been presented using text, graphs, figures and tables. Ethical clearance secured from Mekelle University, College of health sciences, Institutional review board and official letter was obtained from the respective bodies. Information was given to all study participants about the purpose of the study, their right to participate or to terminate at any time if they want was assured and respondents were informed about the confidentiality of information obtained. Then, written informed concent was obtained from each participants before the interview.

### Operational definition

Adequate knowledge: those who scored above the mean on knowledge items and.

Inadequate knowledge: those who scored below mean to knowledge items.

## Results

### Sociodemographic characteristics

Out of the expected 261 respondents in this study, 251 participated with response rate of 96.5%. More than half 147 (58.6%) of the participants were females with the mean age of 34 years (SD = ±9.899). About 53.8% were in age group of 20–30 years. Of all participants majority (93.6%) were registered staff nurses and 6.4% were head nurses. More than a quarter of the participants (38.2%) had 2–4 years of nursing experience but only 12.7% had work experience of more than 10 years. Out of the total participants, 87.6% were degree holders in their level of education and 239 (95.2%) were full-time workers. Eighty-five (33.9%) participants were from medical ward while 28.7% were from pediatric ward (Table 1).

**Table 1:** Socio-demographic characteristics of nurses working in public hospitals in Mekelle City, North Ethiopia, 2015

Variable	Frequency *(N* = *251)*	Percent (%)
Sex		
Male	104	41.4
Female	147	58.6
Age (in years)		
20–30	135	53.8
31–40	49	19.5
41–50	48	19.1
50–60	19	7.6
Level of education		
Degree	220	87.6
Diploma	31	12.4
Work experience (in years)		
<2	73	29.1
2–5	59	23.5
6–9	50	19.9
10 or more	69	27.5
Ever worked in pediatric ward		
Yes	194	77.3
No	57	22.7
Usual rotation		
Days only	74	29.5
Nights only	5	2.0
Rotating shift	172	68.5
Work site		
Emergency department	19	7.6
PICU/AICU	45	17.9
Medical ward	85	33.9
Surgical ward	30	12.0
Pediatric ward/burn unit	72	28.7

### Knowledge of nurses towards pain management

**Fig. 1:**
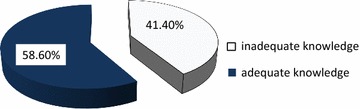
Knowledge of pain management in children among nursing in public hospitals in Mekelle City, North Ethiopia, 2015

Nearly half 121 (48.2%) of the participants answered that nurses provide the most accurate rating whereas only 16 (6.4%) relatives scored the least to accurately rate pain intensity and manage pain. About 88.8% respondent knew that frequently assessing and documenting pain is important whereas 11.2% responded not important for patients who able to communicate. Majority of the participants knew the importance of pain management among; post-operative patients (91.2%), medical (81.7%), patients with trauma (90.4%). But more than half of participant (66.9%) did not know how to manage pain for patients with Glasgow Coma Scale <8 (Table 2). About 58.6% had adequate knowledge about pain management in children (Fig. 1).

**Table 2:** Knowledge on pain management among nurses for hospitalized children, Mekelle City, North Ethiopia, 2015

S. No	Questions	Response	Frequency *(n* = *251)*	Percent
1.	Narcotics on a regular schedule is preferred over ‘PRN’* schedule for continuous pain	Yes	162	64.5
		No	89	35.5
2.	Accurate judge of the intensity of the patient’s pain is the patient	Yes	196	78.1
		No	55	21.9
3.	Distraction by use of music or relaxation decrease feeling of pain	Yes	179	71.3
		No	72	28.6
4.	Increasing narcotic analgesic requirement are signs, patient is becoming addicted.	Yes	195	77.7
		No	56	22.3
5.	Severe chronic pain often need higher dosages of pain medications than acute pain	Yes	173	68.9
		No	78	31.1
6.	Narcotics for pediatric patients can cause respiratory depression	Yes	138	55.0
		No	113	45.0
7.	Analgesics for chronic joint pain cases as needed	Yes	227	90.4
		No	24	9.6
8.	Analgesic for cancer pain patients as needed	Yes	176	70.1
		No	75	29.9
9.	Reports of patient/family, narcotic causing euphoria, should be given a lower dose of the analgesic	Yes	182	72.5
		No	69	27.5
10.	Do children need better attention for managing their pain?	Yes	212	84.5
		No	39	15.5

*PRN* pro re nata/as required

### Factors related to knowledge of nurses for pain management

Logistic regression was used to determine the set of variables which predicted knowledge of pain management. The variables that showed significant association in bivariate logistic regression were: experience in caring for hospitalized children, qualification, usual shift of rotation, lack of availability of pain assessment tool, patient inability to communicate, lack of protocols, experience on caring for hospitalized children, having pain management guidelines, specific protocol, reading guidelines in managing children’s pain, insufficient anesthesia, poor communication of pain assessment, poor documentation of pain assessment and management, sedation interfering, no designated area for charting pain, low priority of pain. Finally sedation interfering with pain assessment and specific protocol remained significant in the multivariate analysis. Those respondents who said yes sedation interfering with pain assessment were 2.7 more likely knowledgeable on pain management for hospitalized children than their counter parts [AOR 2.707 (1.4–5.232)]. In addition to this those nurses who said they had a specific pain management protocol in their institution were 2.159 more likely knowledgeable [AOR 2.159 (1.106–4.212)] (Table 3).

**Table 3:** Socio-demographic and other determinant variables on knowledge of nurses for pain management in public hospitals, Mekelle City, North Ethiopia, 2015 (n = 251)

Characteristics	Knowledge		Odds ratio (95% CI)	
	Inadequate	Adequate	COR	AOR
Qualification				
Diploma	19	12	1	1
Degree	85	135	2.515 (1.162–5.442)*	2.226 (.942–5.258)
Usual shift of rotation				
Days only	40	34	1	1
Nights only	4	1	.294 (.031–2.759)	.275 (.026–2.853)
Rotating shift	60	112	2.196 (1.261–3.823)*	1.755 (.936–3.293)
Patient inability to communicate				
No	45	32	1	1
Yes	59	115	2.741 (1.579–4.757)*	.609 (.304–1.218)
Lack of protocols				
No	36	30	1	1
Yes	68	117	.484 (.274–.856)*	.944 (.439–2.029)
Low priority of pain				
No	47	34	1	1
Yes	57	113	.365 (.212–.629)*	.955 (.457–1.996)
Sedation interfering				
No	59	37	1	1
Yes	45	110	3.898 (2.277–6.674)*	2.707 (1.4–5.232)**
Poor communication of pain assessment				
No	45	32	1	1
Yes	59	115	3.413 (1.920–6.066)*	1.104 (.533–2.286)
Read guidelines for managing children’s pain				
No	60	52	1	1
Yes	44	95	2.491 (1.488–4.171)*	1.252 (.643–2.439)
Specific protocol				
No	66	57	1	1
Yes	38	90	2.742 (1.632–4.608)*	2.159 (1.106–4.212)**

* p ≤ .05, *CI* 95% (confidence interval), *COD* crude odds ratio, *AOD* adjusted odds ratio

** Remained statistically significant in both crude and adjusted odds ratio

## Discussion

A total of 251 nurses were participated in this study, out of these 58.60% nurses had adequate knowledge with some gaps that need consideration. This was almost similar with a study done in Nigeria in which 60% of nurses had knowledge and a study conducted in Bangladesh in which 66.79% nurses had adequate knowledge [13, 14]. On the other this was higher than a study done in Uganda Mulango hospital in which 41% nurses had adequate knowledge [15]. This variation might be because of time gap and different study subjects.

Majority of the participants knew that it is important to manage pain among; post operative patient (91.2%), medical (81.7%), trauma patients (90.4%), and Burn patient (89.6%). These results are reasonable with the fact that these patients need special care for their high pain intensity and most of the time better treatments are given in ICU’s jointly with pain management treatments. But more than half of participant did not know that managing pain for patients with Glasgow Coma Scale <8 is important; this could be due to the nurses misperception on unconsciousness believing unconscious children don’t feel pain since the patient don’t communicate besides being a child. Because of the fact that Pathophysiology of pain is complex and wide array, its management requires multimodal approach; pharmacotherapy (NSAIDs, adjuvant analgesics, opioid) and non pharmacologic ones [16].

Concerning the non-pharmacological management, 71.3% knew distraction of pain by music and relaxation is appropriate in managing children’s pain. In the contrary a study in Hong Kong showed that nurses have inadequate knowledge about both pharmacological and non-pharmacological interventions for pain [17]. This difference might be due to time gap, study area and study design difference.

Those respondents who said yes sedation interfering with pain assessment were more likely knowledgeable on pain management for hospitalized children than others, these showed their genuine response in the area of pain assessment for sedated patients. Fifty-one percent of respondents who have specific protocols in their institution are found to be two times more likely knowledgeable, which is very promising to the institution since the hospitals are public teaching and as well as referral hospitals.

## Conclusion

The study tried to determine the knowledge and factors associated with pain management. Based on the findings the study the following was concluded:

- Majority of nurses have adequate knowledge about the principles of pharmacological and non pharmacological pain managements. However, they lack knowledge on some key issues of opioid analgesics.
- Pain managements of nurses is mostly constrained by; lack of guidelines and specific protocols, inadequate knowledge of the nurses, unavailability of documentation charts, not working in pediatric wards and sedation interfering with pain assessment. These all factors were pertinent for the management of pain in children.

## Abbreviations

AICU: adult intensive care unit
ANA: American Nurses’ Association
NSAID: non steroidal anti inflammatory drugs
PICU: pediatric intensive care unit
PRN: pro re nata/as needed
VAS: visual analog scale
